# Sugar Intake: Are All Children Made of Sugar?

**DOI:** 10.3390/life11050444

**Published:** 2021-05-14

**Authors:** Lucia Diani, Maria Luisa Forchielli

**Affiliations:** 1 Dietetics, Morgagni Pierantoni Hospital, 47121 Forli, Italy; lucia.diani@auslromagna.it; 2 DIMEC, University of Bologna, 40138 Bologna, Italy; 3 Health Science and Technologies Interdepartmental Center for Industrial Research (CIRI-SDV), University of Bologna, 40100 Bologna, Italy

**Keywords:** children, nutrition, health-diet, celiac disease, sugar-free

## Abstract

Introduction: A healthy diet is characterized by a variety of food and a balanced energy intake, which should accompany every human being since early childhood. Unfortunately, excessive consumption of protein, fat, and lately sugar are very common in developed countries. Sugar intakes are not easily quantifiable and comparable among subjects. Therefore, we decide to analyze dietary patterns in children of different ages and diets (with and without gluten) using a food and nutrient database and a new application called the “Zuccherometro”. Patients and methods: This is a descriptive observational study conducted among children that are recruited consecutively either during a pediatric evaluation or through a school survey. Sociodemographic, nutritional and anthropometric data, degree of physical activity, and presence of medical conditions are collected. Dietary intake data are obtained by a 24 h recall diet. Results: The study analyzes 400 children: 213 girls and 187 boys. The majority of children (70.7%) are in normal weight range with similar extreme values (6.5% obese and 6.7% underweight). Celiac disease is diagnosed in 186 children. Caloric intakes are in line with the recommendations in all age-distributed groups with the exception of adolescents (11–17 years old), whose caloric intake is lower than recommendations. Protein intakes, on the contrary, are always exceeding recommendations and are significantly elevated in preschool children, (more than three times the population reference intakes). As for sugar intakes, all the children except the 11–17 years adolescents exceed the recommended cut off of 15% of daily calories. The same trend is obtained using the “Zuccherometro” that shows different percentages of age-stratified children exceeding the reference values: 1–3 years, 59% of children; 4–6 years, 68%; 7–10 years, 39.8%; 11–14 years, 25.5%; 15–17 years, 24.5%. The sugar load consists of both natural or added sugars (fructose and lactose) in food or beverages. Sugar intakes are more generously consumed by all age-stratified controls than by celiac children with the exception of the youngest ones (1–3 years old) and male adolescents. Conclusion: Since high sugar intakes are constantly accompanying children during their growth, important dietary education and coordination between families and institutions are mandatory.

## 1. Introduction

Healthy diets are characterized by a variety of food and a balanced energy intake and they should accompany every human being since early childhood. Nutrition is one of the environmental factors that can affect pediatric growth and neurodevelopment along with demographic, socioeconomic, and behavioral aspects [1,2]. The literature offers several examples of public health problems interconnected with nutrition. Overweight/obesity is one of these examples. It affects children worldwide and exposes them to poor physical and psychological health [3,4]. Obese children are likely to stay obese into adulthood and more likely to develop noncommunicable diseases such as diabetes and cardiovascular diseases at a younger age with a reduced life expectancy [5,6]. A high consumption of junk food, energy-dense food, and a high intake of saturated fat, sugar, and salt, associated with low levels of physical activity, are the main causes of obesity as well as many other diseases [7]. Sugar assumption has increased over time and recommendations at the international and national levels were released to contain its intake both in children and adults (<10% of daily energy intake by the World Health Organization (WHO) and <15% by the Italian population (LARN)] [8,9]). Recent Italian national surveys reported a 21% daily energy intake of free sugar in children and adolescents [10,11]. Specific groups such as the celiac disease patients can be exposed to high intake of sugar, in relation to the increased consumption of processed foods [12]. However, data cannot be used in a generalized manner due to diversity in nutrition software analysis. The recent release of a software called “Zuccherometro” computing the amount of sugar intake and confronting it to common food was believed to be an opportunity to run a healthy survey in a topic of emerging needs. For this reason, we decide to analyze dietary patterns in children of different ages as a primary endpoint and in children adhering to different diet patterns such as gluten-free using both food and nutrient database and the Zuccherometro, which may be an easy tool for families. Testing the software feasibility is also an opportunity to optimize nutrition educational tools which are scarce [13].

## 2. Materials and Methods

### 2.1. Study Design

This is a descriptive observational study in children during a routine visit or through a school survey, done during a food educational project. Families received a questionnaire which included the below collection of data. Children were classified by age and health status (presence of disease, malformation, use of drugs). The protocol was approved by the Ethics Committee of the University Hospital (75762/4/3/19-38/2017/O/Oss-) and the study applied the Helsinki Declaration. After providing explanation of the study and guarantying anonymity, parents signed an informed consent.

### 2.2. Collection of Data

Sociodemographic, nutritional, degree of physical activity, and anthropometric data were collected along with type of disease and consequent medications, if present. Anthropometric data (weight (kg), height (cm), weight-height-body mass index (BMI), waist circumference (cm), hip circumference (cm) and wrist circumference (cm)), if available, were classified according to the Italian growth standard and their nutritional status evaluated [14]. The body mass index (BMI) was calculated and classified according to the World Health Organization (WHO) [15].

### 2.3. Dietary Intake Data

Parents along with their children filled in the questionnaire instructed by dietitian or trained staff on a 24 h recall diet. Recall diets were recorded considering whether they occurred in a week or week-end day, at school, or at home. If the previous day was not representative of the child’s food habits (e.g., school trip, family event), the day before was recorded instead. Food dressings, drinks (plain or carbonated water or soda), and sweets were also recorded. Food portions were determined using common kitchen tools such as measuring spoons or food scale. The collected information was analyzed using the Atlante Alimentare Scotti-Bassani, Istituto Europeo d’Oncologia (IEO) data center, and the “Zuccherometro”, an internet tool specifically planned to compute sugar content in drink and food with added sugar including junk food (i.e., processed food like biscuits, chocolate, candys, and so on) [16,17]. The Zuccherometro tool also shows recommended intakes of free added sugars based on each subject’s age and sex.

### 2.4. Statistical Analysis

Data are summarized based on distribution as means and standard deviations (SD) or medians and ranges (min–max).

The patient’s dietary habits were compared to the LARN recommendations; the children were stratified into age-based groups from 1 year to 17 years old [18]. Sugar amounts were computed applying the Zuccherometro at the children’s dietary habits and these amounts were compared to the recommended quantities. We used Student test to compare all collected variables within and between groups by *t* test.

## 3. Results

### 3.1. Population Characteristics

The study analyzed 400 Caucasian children (age range 1–17 years, median 10 years, mean 11 ± 6 years), 213 girls and 187 boys. Table 1 shows the baseline characteristics of the participating children with a similar gender distribution (53% females vs. 47% males). The majority of children (70.7%) are in normal weight range with similar distribution in the two extremes of the growth curve (6.5% obese and 6.7% underweight).

The children referred to the hospital were in follow up for celiac disease. Two populations seem to be homogenous about anthropometric values, physical activity, and dietary intakes (Table 2).

### 3.2. Daily Dietary Intakes in the Different Age Groups

After stratifying the children into five age groups (1–3-year-olds; 4–6-year-olds; 7–10-year-olds; 11–14-year-olds; 15–17-year-olds), we compare their energy intakes with the mean LARN recommendations based on age and gender. It appears that caloric intakes are in line with the recommendations in all groups with the exception of the 11–17 years old adolescents, in which caloric intakes are lower than recommendation. Protein intakes, on the contrary, are always exceeding recommendations and are significantly elevated in preschool children, (3.8 times and 3.2 times over PRI value in first and second group). As for sugar intakes, all the children except the 11–17 years old adolescents exceed the recommended cut off of 15% of calories. In the first two groups, sugar intakes are exaggerated.

The same trend is obtained using the “Zuccherometro”, with the following results:

(1) 1–3 years, 59.09% sugar-meter values higher than the reference;
(2) 4–6 years, 67.9% higher values;
(3) 7–10 years, 39.81%;
(4) 11–14 years, 25.49%;
(5) 15–17 years, 24.53%.

In general, cocoa powder, chocolate, hazelnut cream, and biscuits used at breakfast are equally distributed in the groups. However, soda drinks and especially fruit juices seem to be the most represented in all the groups.

Table 3 (a,b) show preferences of children with celiac disease and controls toward sugary food and drinks. Table 4 summarizes sugar contents in the diets of controls and celiac children based on age groups. Table 5 shows data relating to the other macronutrients.

### 3.3. Dietary Intakes by Age and Sex

After stratifying children based on age and gender (Table 4, Table 5 and Table 6), both males and females in the oldest age groups ingest less calories than recommended. Protein intakes, on the contrary, are higher than recommendations in all groups regardless of gender. Celiac children, however, tend to have more pronounced protein intakes over the years when compared to the controls and especially in the 15–17-year-old adolescents (*p* < 0.01) and male adolescents. In addition, lipid intakes appear generously present in the 15–17-year-old celiac group (*p* < 0.01), but this time celiac males as the control introduce less lipids. Both groups have a lower intake of fibers over the years. Celiac females, on the opposite, are the ones who show increasing intakes of lipids and fibers over the years, while controls have constant intakes. Celiac males (7–10 years) have low intakes of calcium (average 470 mg vs. 670 mg/day in controls, *p* < 0.002) and iron (average 6.4 vs. 9.4 mg/day in controls, *p* < 0.002). Both controls and celiac children seem to have a decreasing trend in free sugar intakes throughout the age categories. The youngest celiac group (1–3 years) has the worst free-sugar intakes when compared to the same age group in controls (*p* < 0.05) and all the other groups.

In the first age group biscuits and yogurt are the most contributors of high sugar intake (>80% of sample), in the second group juice and biscuits (>60%) and cocoa for milk (>60%), in the third juice and sweet snack (>70%), while in the fourth and fifth groups sugary drinks (juice, tea, cola) (>50% and >80%, respectively).

Revaluating the simple sugar intakes based on the Zuccherometro, females until 10 years of age have an excessive consumption with a sharp decline thereafter (Table 7). Unlike females, males exceed sugar intakes more than 50% from 1 to 5 years of age but later contain them at approximately 30% even in adolescence.

## 4. Discussion

In this study we address nutritional intakes in children with a particular emphasis toward the ingestion of simple sugars, which can easily be quantified and compared by a recently designed internet tool, the Zuccherometro. The analysis covers general pediatric population and children with celiac disease during gluten exclusion which is considered a dietary regimen potentially hazardous for excessive sugar intake [19]. We found that younger children tend to exceed in caloric intakes when compared with reference values while older children seem to hardly reach the target. Younger children also ingest higher amounts of proteins and simple sugars. As they grow, children show great attention to diets especially after 14 years of age and in females, the trend is in line with other data [20].

Protein intakes are excessive in all children (celiac and controls) and especially in the 1 to 6-years-olds. They consume 3.69 g/kg/d in the age range of 1 to 3 and 3.05 g/kg/d in the 4 to 6-year-olds. Both intakes, which are over 200% higher than the recommendations, are similar to the amounts described in another recent Italian study in which the 1–3-year-old children had an average of 3.5 g/kg/d [10]; the amounts were the same in the European data [21]. When computed as percentage of total caloric intakes, our children have protein intakes within the normal range. This observation stresses the need to express protein data as grams to children’s weights instead of percentages of total calories [22]. This process will avoid erroneous perceptions and calculations when planning diets.

Almost three quarters (67%) of the children under 11 years of age have simple sugars intake greater than the recommended 15% cut off of daily energy intake. Toddler celiac males have the highest sugar intakes. The sugar load consists of both natural sugars (fructose and lactose) and added sugars or sugary foods. Added sugar food and beverages are indeed the main culprit for exaggerated intakes in the 1–3-year-old children and the 4–6-year-olds [23] (both groups exceeding by 59% and 68% respectively the reference values according to the Zuccherometro). Fiber intakes on the other hand do not reach the recommended ratio of 8.4 g/1000 Kcal, further aggravating the glycemic index balance. The age range of 4 to 10 years old is the only one approaching the suggested fiber intake. The combined efforts of parents and school personnel may have inspired the children to act properly, but an extra effort seems necessary in the early ages, especially in families with celiac patients even though the small number of toddler celiac males (4 subjects) needs a larger sample size [24].

The sugar intakes of our children differ from that reported in a European multicenter study in which the 2–9-year-old children from Italy had an average 13% intake. Food groups that contributed substantially to free sugar intakes are similar [25]. The majority of children eat mostly packaged and ready-for-consumption food products which are equally divided into biscuits and snacks. Breakfast and snacks are the main opportunities for sugar introduction with a 60% concentration in the morning, 35% in snacks, and only 5% at other meals [26]. Sugary drinks are, on the opposite, present throughout the day distributed mainly at snacks (65%), followed by dinner (20%), lunch (10%), and finally breakfast (5%). It was particularly unexpected that the excessive intake of sugary drinks occurred for the most part at dinner rather than during snacks and breakfast. The 30.9% (113 children) of the controls and only 25% (46 children) of the celiac group consume sugary drinks (113 children) at dinner. It is possible that this observation is biased by data collection through the food-frequency questionnaires, but the current trend of a decrease water intake substituted by other fluids slowly but surely is taking control of families’ eating patterns. We frequently find parents who mention to “edulcorate” water with fruit syrups or similar sweeteners or soda because otherwise their children will not drink it. This step can be a prelude to addiction which is already proven in animal study [27]. Lenoir et al. found that intense sweetness is far more potent than cocaine rewards in rats and speculated that addiction is possible in diets at high load of sugar [28]. Whether it could be considered children’s addiction or a hidden challenge for parents in educating their children can be matter of investigation.

A possible reason for these excessive “rich” diets may be the sequence of food choices between home and school with repetition of biscuits with milk or yogurt or juices at breakfast and mid-morning. These sequences are common in toddlers and children attending nursery and kindergarten. As children get older, they are at risk of extra intakes during recess times in which sweet snacks, juices, ready-to drink teas, soda, and cocoa milk are easy and quickly ingestible food. Recess times multiply in middle school with up to 80% of sugar intake represented by these drinks [29]. Among these drinks, fruit juices are the more frequent in all the groups. Considering that 100 mL of juice contains more sugar and less satiety effect (on average 2.5 (12.5 g) teaspoons of sugar and 0 g of fiber compared to 2 (10 g) of sugar and 2.5 g of fiber in 100 gr of fresh fruit), this simple and resolvable aspect should be used to raise parents’ and institutions’ awareness in the attempt to synchronize their efforts. Furthermore, 30% of the sample takes at least one fruit juice (200 mL) per day which is about 25 g of sugars.

Children who consume classic sugar-free rather than sugar-coated breakfast cereals reduce simple sugar intake by approximately 10 g per 100 g of product. Likewise, two slices of bread with jam (10 g) provide the same quantity of simple sugars as 5 shortbread biscuits but with lower caloric and higher fiber content which increases satiety. Prolonged satiety is also obtained by the higher number of chews required to fragment bread instead of biscuits allowing to reach prolonged satiety in the first case.

When different diet patterns are considered, children on free diets seem to have an average consumption of 2.3 products containing simple sugar and 0.5 soft drinks per day. On the contrary, children on a gluten-free diet seem to pay more attention to diet quality as they have an average of 1.8 simple sugar food items and 0.36 soft drink beverages per day. The sugar intakes of our celiac adolescents are more limited in comparison to the findings of the Babio et al. study [12]. In general, cocoa, chocolate, hazelnut cream, and biscuits for breakfast are equally distributed in the groups. However, the preference of children on gluten-free diet is towards artisanal and homemade products except for ice cream and hazelnut cream. These results may indicate that people with celiac disease and their families may reach higher nutrition knowledge and behavior. Their recurrent checking on food ingredients and their tendency to look for artisan and homemade food allows them to make better choices. Moreover, children with celiac diseases are under a follow up which may be an opportunity for them and their families to discuss nutritional issues. Another point to be verified is whether the Zuccherometro tool may not be taking into consideration some of the gluten-free food, even though most of the classified items are also permitted to celiac children and considered in the present analysis. Statistical analysis shows that the difference between the content of simple sugars in the two groups is not statistically significant with the exception of toddler celiac males. This may be an effect of the small numbers after stratification. No more conclusions can be drawn, but we feel more research should be done to explain the high values found through the Zuccherometro tool between celiac and control groups.

## 5. Conclusions

This study stresses the need for dietary education and for coordination between families and institutions to reduce sugar intake in children. This new application can be an easy tool for families.

Our data suggest that the diagnosis of celiac disease is likely to push families and children to pay greater attention to food quality. This may lead to a more controlled diet, especially in terms of simple sugars. The medical follow-ups also provide opportunities for nutrition education and could reinforce the families’ efforts. The second learning point is that early ages are more exposed to excess intakes of sugary food. Daily meal patterns should be urgently reassessed.

## Figures and Tables

**Table 1:** Demographic data of the children.

	M	F	Total
**Sex**	187	213	400
**Obese**	15	11	26 (6.5%)
**Overweight**	34	30	64 (16%)
**Normal range**	127	156	283
**Underweight**	11	16	27 (6.7%)
**Celiac disease**	76	110	186
**Controls**	111	103	214
**Physical activity (%child)**	34%	29%	
**Weekend/Week**	15%/85%	18%/82%	
**School/Home**	33%/67%	30%/70%	

**Table 2:** Anthropometric characteristics and nutrition intakes of children.

	Celiac Children (186)	Controls (214)
**Weight (kg)**	36.98	38.87
**Height (cm)**	138.15	134.44
**BMI**	18.09	18.76
**Kcal/d (mean ± SD)**	1776 ± 461	1765 ± 415
**Protein (g/d; mean ± SD))**	66.5 ± 24	66.4 ± 19
**Carbohydrates (g/d; mean ± SD)**	234.3 ± 71	238 ± 64
**Sugar (g/d; mean ± SD)**	75 ± 32	73 ± 29
**Lipids (g/d; mean ± SD)**	67.8 ± 23	65 ± 22

**Table 3:** (**a**) Distribution of “sugary food” in the children. (**b**) Distribution of “sugary drink” in the children.

(a)			
	Total	Controls	Celiacs
**Biscuits**	42	35	7
**Shortbread**	179	105	74
**Snack**	112	71	41
**Croissant**	22	14	8
**Commercial brioche**	10	3	7
**Cake**	24	8	16
**Hazelnut cream**	137	78	59
**Homemade ice cream**	33	29	4
**Ice cream**	27	9	18
**Wafer**	25	0	25
**Cocoa powder**	24	17	7
**Yogurt**	82	47	35
**Cereals**	49	31	18
**Chocolate**	76	76	30
**Total**	842	493	349
**(b)**			
	**Total**	**Controls**	**Celiacs**
**Juice**	121	78	43
**Ice-tea**	32	16	16
**Coke**	11	8	3
**Other beverages**	17	11	6
**Total**	181	113	68

**Table 4:** Added sugar intake divided by ages groups.

	LARN (g * ± SD) (%)	Mean (g * ± SD) (%)	Median (Range) (g) (%)	Controls Mean (g * ± SD) (%)	Celiacs Mean (g * ±SD) (%)
**Free Sugar** **1–3 years (number of children 45; controls 27/celiacs 18)**	40.5 ± 12 <15 (STD)	65.4 ± 21.2 ^^^ 19.4 ± 5.5	62.6 (24–109) 19 (9.2–32.6)	70.28 ± 20.8 ### 19.4 ± 5.7	71.8 ± 21.2 §§§ 20.3 ± 5.4
**Free Sugar** **4–6 years (*n* 52; 28/24)**	56 ± 15 <15 (STD)	81.3 ± 22.6 ^^^ 19.9 ± 4.6	78.8 (44–143.3) 19.3 (12.2–38.2)	78.7 ± 17.4 ### 19.8 ± 3.9	79.5 ± 24.2 §§§ 20.7 ± 4.5
**Free Sugar** **7–10 years (*n* 131; 69/62)**	72.7 ± 21 <15 (STD)	79.2 ± 30.6 ^ 17.4 ± 6.3	78.7 (18.2–150.8) 16.5 (6.2–34.4)	81.8 ± 29.4 # 17.7 ± 5.7	79.8 ± 29.7 18.2 ± 6.5
**Free Sugar** **11–14 years (*n* 86; 43/43)**	95 ± 40 <15 (STD)	68.7 ± 31.9 ^^^ 14.4 ± 6	62 (10.6–167.9) 13.9 (3–35)	71.5 ± 32.9 ### 14.5 ± 6.5	65.2 ± 30.3 §§§ 14.1 ± 6.2
**Free Sugar** **15–17 years** **(*n* 86; 47/39)**	99 ± 45 <15 (STD)	72.2 ± 39.3 ^^^ 14.9 ± 6	61.5 (12.9–244.4) 14.2 (3.2–33.5)	65.7 ± 31.8 ### 15.1 ± 8.9	77.2 ± 44.6 § 15.2 ± 5.8

SDT: suggested dietary target consisting of <15% of total daily energy intake. *: grams of free sugars were extrapolated from the daily average age range energy recommendations based on the LARN. Statistically significant differences of overall sample measurements from recommended intake at *p* < 0.05 (^), *p* < 0.001 (^^^); celiac from recommended intake at *p* < 0.05 (§), *p* < 0.001 (§§§); controls from recommended intake at *p* < 0.05 (#), *p* < 0.001 (###). No statistical differences were found comparing controls with celiac children.

**Table 5:** Macronutrients and energy intake divided by age group.

	LARN	Mean ± SD	Median (Range)	Controls (Mean ± SD)	Celiacs (Mean ± SD)
**Age** **(1–3 years)** ***n* 45**		***n* 45**		***n* 27**	***n* 18**
**Kcal**	male 870–1390 female 790–1280	1347.1 ± 230 **	1311.5 (719.6–1961.6)	1460.2 ± 160 ###	1410.5 ± 199.6 §§§
**Protein g** **g/Kg weight**	11 (AR)–14 (PRI) 0.82 (AR)–1 (PRI)	53.5 ± 14 4 ± 1.3 ***	53.2 (20.3–78.4) 3.69 (1.46–6.56)	56.8 ± 13.6 3.6 ± 1.3 ###	53.4 ± 14.2 3.8± 1.4 §§§
**Lipid g** **%**	- <40 (RI)	51.4 ± 14 33.6 ± 7	47.9 (30–89.2) 34 (20–49)	55.7 ± 13.9 33.3 ± 7.1 ##	54.8 ± 13.6 34.5 ± 6.1 §§
**Fiber g** **g/1000 Kcal**	- 8.4 (AI)	9 ± 5 6.8 ± 3 **	8.1 (1.9–26.3) 6 (1.7–20.1)	10.9 ± 4.6 7.5 ± 3.5 #	9 ± 4.6 6.5 ± 3.2 §§
**Age** **(4–6 years)** ***n* 52**		***n* 52**		***n* 28**	***n* 24**
**Kcal**	male 1470–1640 female 1350–1520	1641 ± 328 **	1613.2 (1017.5–2565)	1611.5 ± 240 #	1539.3 ± 199.6
**Protein g** **g/Kg weight**	16 (AR)–19 (PRI) 0.76 (AR)–0.94 (PRI)	61.2 ± 18 3.1 ± 1 ***	61.6 (23.9–103.6) 3.05 (1.17–75.6)	60.2 ± 14.9 3.2 ± 0.8 ###	53.3 ± 19.7 2.8 ± 1.1 §§§
**Lipid g** **%**	- <35 (RI)	61.2 ± 17 33 ± 7	60.8 (29.4–107.3) 33 (20–52)	62.1 ± 13.9 33.4 ± 6.5	58.7 ± 15.4 33.9 ± 6.6
**Fiber g** **g/1000 Kcal**	- 8.4 (AI)	13.4 ± 4.5 8.2 ± 2.5	13.1 (4.2–25.6) 8.1 (3.9–15.6)	12.9 ± 4.9 8.2 ± 3.1	12.5 ± 4.2 8.2 ± 2.5
**Age** **(7–10 years)** ***n* 131**		***n* 131**		***n* 69**	***n* 62**
**Kcal**	male 1750–2300 female 1620–2090	1825 ± 334 ***	1817.9 (1014–2570.6)	1843.8 ± 336 #	1765.2 ± 326.2 §§§
**Protein g** **g/Kg weight**	25 (AR)–31 (PRI) 0.81 (AR)–0.99 (PRI)	67 ± 17.6 2.2 ± 0.8 ***	68.4 (26.3–110.6) 2.22 (0.63–4.03)	69.4 ± 17 2.5 ± 1.1 ###	64.9 ± 18.1 2.4 ± 0.9 §§§
**Lipid g** **%**	- <35 (RI)	67.6 ± 19 32.6 ± 7 **	65.9 (24.1–124) 32 (18–54)	67.9 ± 19.1 32.2 ± 6.8 ##	64.2 ± 18.8 32.1 ± 6.9 §§
**Fiber g** **g/1000 Kcal**	- 8.4 (AI)	14.7 ± 5 8 ± 3	14 (5.5–27.7) 7.8 (2.8–14.4)	14.4 ± 5 7.9 ± 2.7	14.9 ± 5.3 8.2 ± 3 ^^
**Age** **(11–14 years)** ***n* 86**		***n* 86**		***n* 43**	***n* 43**
**Kcal**	male 2440–2960 female 2210–2490	1907 ± 401 ***	1834.4 (1150.2–3413)	1949.9 ± 454 ###	1839.2 ± 312 §§§
**Protein** **g/Kg weight**	39 (AR)–48 (PRI) 0.78(AR)–0.96(PRI)	73 ± 23 1.6 ± 0.7 ***	69.1 (34.8–161) 1.46 (0.54–3.76)	73.8 ± 21.9 1.6 ± 0.6 ###	72.5 ± 2.9 1.7 ± 0.7 §§§
**Lipid g** **%**	- <35 (RI)	71 ± 24 33 ± 7.6	68.1 (21.7–157.6) 32 (12–57)	70.6 ± 26.3 31.5 ± 6.5	69.7 ± 19.5 33.2 ± 7.3
**Fiber g** **g/1000 Kcal**	- 8.4 (AI)	14 ± 5 7.6 ± 2.6	13.5 (5.6–36.2) 6.9 (2.9–15.3)	14.8 ± 5.2 7.6 ± 2.9	14 ± 5.4 7.4 ± 2.9 §
**Age** **(15–17 years)** ***n* 86**		***n* 86**		***n* 47**	***n* 39**
**Kcal**	male 3110–3260 female 2510	1948 ± 626 ***	1806.7 (1119.9–4423)	1794.7 ± 422 ###	2011. 5 ± 702 §§§
**Protein g** **g/Kg weight**	45 (AR)–56 (PRI) 0.75(AR)–0.91(PRI)	73.5 ± 28.6 1.3 ± 0.4 ***	67.2 (29.2–207) 1.27 (0.45–2.86)	66.5 ± 23.3 1.1 ± 0.5 ##	75.8 ± 31.8 1.4 ± 0.5 §§§^^
**Lipid g** **%**	- <35 (RI)	74 ± 30 33.3 ± 7	69.8 (25.4–202.1) 33 (15–47)	66.1 ± 24.1 31.9 ± 8.9	81.7 ± 31.4 36 ± 6.2 ^^
**Fiber g** **g/1000 Kcal**	- 8.4 (AI)	14.1 ± 7 7.3 ± 3 ***	13.5 (3.7–42.8) 6.9 (2.4–15.8)	13.8 ± 6.6 7.4 ± 4.3	15.3 ± 8 7.9 ± 3.9

AI: Adequate intakes. PRI: population reference intakes. AR: average intakes. RI: upper level of reference intake value. When recommended intakes were shown as ranges, an average value was considered in the statistical analysis. Statistically significant differences of overall sample measurements: from recommended intakes at *p* < 0.01 (**), *p* < 0.001 (***); celiac from recommended intakes at *p* < 0.05 (§), *p* < 0.01 (§§), *p* < 0.001 (§§§); controls from recommended intakes at *p* < 0.05 (#), *p* < 0.01 (##), *p* < 0.001 (###); from controls compared to celiac intakes at *p* < 0.01 (^^).

**Table 6:** Macronutrients and energy intakes in celiac and controls stratified by age and gender.

Males	Reference Value	Mean	SD	Controls (Mean; SD)	Celiacs (Mean; SD)
**Kcal**	**A**. 870–1390 **B**. 1470–1640 **C**. 1750–2300 **D**. 2440–2960 **E**. 3110–3260	1372 1668.6 1888 1933.7 2301.8	179.9 370.1 341.9 385.6 767.8	1340.3 ± 179.7 1612 ±313.3 1930.3 ± 341.6 § 1921.4 ± 410.4 1998.8 ± 449.1 §§	1506.5 ± 117.7 1624.4 ± 398.4 1797.6 ± 357.4 1980.7 ± 296.5 ° 2496.3 ± 942.3 #
**Protein g**	**A**. 11 (AR)–14 (PRI) **B**. 16 (AR)–19 (PRI) **C**. 25 (AR)–31 (PRI) **D**. 39 (AR)–48 (PRI) **E**. 50 (AR)–62 (PRI)	54.9 63.5 70.4 75.5 89.8	12.9 19.5 16.9 20.1 35.1	55 ± 13.3 58.9 ±17.3 73.8 ± 16.1§ 72.3 ± 19 77.7 ± 23.5 §§	54.5 ± 13.2 56.8 ± 19.8 69 ± 18 81.6 ± 23 95.8 ± 4.8 #
**Free Sugar %**	**A**. <15 (STD) **B**. <15 (STD) **C**. <15 (STD) **D**. <15 (STD) **E**. <15 (STD)	19.7 20.2 16.9 14.5 14.6	6.3 5.3 5.6 5.5 7.3	18.8 ± 6.6 19.6 ± 4.4 18.5 ± 5.7 15.8 ± 5.9 13.1 ± 7.2 §§	23.9 ± 2.9 *$ 21.6 ± 5.1 16.6 ± 6.1 14 ± 4.8 15.7 ± 7.2
**Lipid %**	**A**. 35–40 (RI) **B**. 20–35 (RI) **C**. 20–35 (RI) **D**. 20–35 (RI) **E**. 20–35 (RI)	33.2 27 33.3 30.2 31.3	2.8 5.7 3.5 2.8 4.9	32.5 ± 6.6 33.7 ± 6.5 32.7 ± 6 30.4 ± 5.9 29.4 ± 7.9	36.5 ± 1.9 32.1 ±5.9 33.1 ± 5.8 31.5 ± 5.9 33.3 ±7.1
**Fibers g**	**A**. 8.4/1000 Kcal **B**. 8.4/1000 Kcal **C**. 8.4/1000 Kcal **D**. 8.4/1000 Kcal **E**. 8.4/1000 Kcal	7.1 8.2 8.2 7.3 6.7	3.9 2.8 2.7 2.4 2.7	6.9 ± 4.2 8.5 ± 3.5 8. 3 ± 2.8 § 7.5 ± 2.2 7.1 ± 4.4	7.7 ± 3 7.7 ± 1.8 7.9 ± 2.8 7.1 ± 2.7 7.1 ± 3.3
**Females**					
**Kcal/d**	**A**. 790–1280 **B**. 1350–1520 **C**. 1620–2090 **D**. 2110–2490 **E**. 2510	1394.4 1606.4 1757.5 1889.5 1696.3	269.3 268.5 314.3 412.7 331.3	1277.3 ± 312.5 1610 ± 232.9 1757.3 ± 311.2 1977.3 ± 499.5 1643.6 ± 333.9	1375.6 ± 215.9 1454.2 ± 251 1731.6 ± 293.1 1765.8 ± 299.7 1795.9 ± 434
**Protein** **(g/d)**	**A**. 11 (AR)–14 (PRI) **B**. 16 (AR)–19 (PRI) **C**. 25 (AR)–31 (PRI) **D**. 39 (AR)–48 (PRI) **E**. 40 (AR)–50 (PRI)	52.1 58.3 63.4 71.6 61.9	14.42 16.2 17.8 25.5 15.1	51.3 ± 14.3 63.3 ± 5.2 65 ± 17 75.2 ± 24.7 58.2 ± 19.7	52.9 ± 15.2 49.9 ± 19.8 60.6 ±17.5 67.8 ± 23.5 66.9 ± 21.7
**Free Sugar (%)**	**A**. <15 (SDT) **B**. <15 (SDT) **C**. <15 (SDT) **D**. <15 (SDT) **E**. <15 (SDT)	19.1 19.5 17.8 14.3 15.2	4.8 3.4 7 6.4 4.5	19.2 ± 4.3 20.4 ± 2.6 16.9 ±5.7 13.7 ±3.4 16.6. ± 9.8	19 ± 5.6 19.8 ± 3.7 19.8 ± 6.6 14.1 ± 6.9 15.1 ± 5.3
**Lipid** **(%)**	**A**. 35–40 (RI) **B**. 20–35 (RI) **C**. 20–35 (RI) **D**. 20–35 (RI) **E**. 20–35 (RI)	33.9 32.9 31.8 34.4 34.7	7.4 1.4 7.9 7.9 6.4	34± 8 32.9 ± 6.8 31.7 ± 7.5 32.5 ± 6.9 33.8 ± 9.5	33.8 ± 7 35.7 ± 6.9 31.1 ± 7.8 34.2 ± 7.9 37.2 ± 5.5
**Fibers** **(g)**	**A**. 8.4/1000 Kcal **B**. 8.4/1000 Kcal **C**. 8.4/1000 Kcal **D**. 8.4/1000 Kcal **E**. 8.4/1000 Kcal	6.5 9.6 7.9 7.8 7.8	2.8 2.4 3.1 2.9 5.	6.9 ± 2.4 7.4 ± 2.3 7.5 ± 2.5 7.7 ± 3.4 7.7 ± 4.2	6.1 ± 3.3 8.7 ± 2.9 8.4 ± 3.3 7.6 ± 2.9 8.2 ± 4.2

**A**: 1–3 years age range. Total children 45 with 27 controls (15 males and 12 females) and 18 celiacs (6 males and 12 females). **B**: 4–6 years age range. Total children 52 with 28 controls (20 males and 8 females) and 24 celiacs (12 males and 12 females). **C**: 7–10 years age range. Total children 131 with 69 controls (36 males and 33 females) and 62 celiacs (30 males ad 32 females). **D**: 11–14 years age range. Total children 86 with 43 controls (20 males and 23 females) and 43 celiacs (16 males and 27 females). **E**: 15–17 years age range. Total children 86 with 47 controls (20 males and 27 females) and 39 celiacs (12 males and 27 females). PRI: population reference intake. AR: average reference intake. SDT: suggested dietary target. RI: reference intake range. Statistically significant differences of overall sample measurements: from comparisons between male and female children in the A group at *p* < 0.05 (*), in the D groups *p* < 0.05 (°), in the E group *p* < 0.05 (#); from comparisons between male and female controls in group C at *p* < 0.05 (§), in the E group *p* < 0.001 (§§); from comparisons between controls and celiac in the same age and gender categories at *p* < 0.05 ($).

**Table 7:** Percentage of children with exceeding sugar intakes.

	Females (*n* 213)	Female Controls (*n* 103)	Female Celiacs (*n* 110)	Males (*n* 187)	Male Controls (*n* 111)	Male Celiacs (*n* 76)
**Age 1–3 years (%)** **(*n* 45)**	65.2 (*n* 24)	75 (*n* 12)	63.6 (*n* 12)	52.4 (*n* 21)	35.3 (*n* 15)	75 (*n* 6)
**Age 4–6 (%)** **(*n* 52)**	69.4 (*n* 20)	33.3 (*n* 8)	61.1 * (*n* 12)	66.7 (*n* 32)	44.4 (*n* 20)	61.1 * (*n* 12)
**Age 7–10 (%)** **(*n* 131)**	44 (*n* 65)	59.2 * (*n* 33)	34.8 (*n* 32)	35.8 (*n* 66)	55.2 * (*n* 36)	12.5 (*n* 32)
**Age 11–14 (%)** **(*n* 86)**	21.3 (*n* 50)	51.7 * (*n* 23)	18.2 (*n* 27)	31.7 (*n* 36)	34.6 (*n* 20)	33.3 (*n* 16)
**Age 15–17 (%)** **(*n* 86)**	16.1 (*n* 54)	31.6 (*n* 27)	20 (*n* 27)	36.4 (*n* 32)	28.6 (*n* 20)	50 * (*n* 12)

*n*: number of patients. %: Percentage of excessive sugar intake in comparison with the required age-specific daily intake Statistically significant differences of overall sample measurements: from comparisons between male celiacs and controls or between female celiacs and controls at *p* < 0.05 (*).

## Data Availability

Not applicable.
